# Progression independent of relapse activity and relapse-associated worsening in seronegative NMOSD: an international cohort study

**DOI:** 10.1007/s00415-025-13064-6

**Published:** 2025-04-14

**Authors:** Pakeeran Siriratnam, Saif Huda, Anneke Van der Walt, Paul Sanfilippo, Sifat Sharmin, Yi Chao Foong, Wei Zhen Yeh, Chao Zhu, Samia J. Khoury, Tunde Csepany, Barbara Willekens, Masoud Etemadifar, Serkan Ozakbas, Petra Nytrova, Ayse Altintas, Abdullah Al-Asmi, Cristina Ramo-Tello, Guy Laureys, Francesco Patti, Dana Horakova, Matteo Foschi, Cavit Boz, Pamela McCombe, Recai Turkoglu, Izanne Roos, Jeannette Lechner-Scott, Tomas Kalincik, Vilija Jokubaitis, Helmut Butzkueven, Mastura Monif

**Affiliations:** 1 Department of Neuroscience, Central Clinical School, Monash University, Melbourne, VIC Australia; 2 Department of Neurology, Alfred Health, Melbourne, VIC Australia; 3 Department of Neurology, Walton Centre NHS Foundation Trust, Liverpool, UK; 4 Department of Neurology, Neuroimmunology Centre, The Royal Melbourne Hospital, Parkville, VIC Australia; 5 CORe, Department of Medicine, University of Melbourne, Melbourne, VIC Australia; 6 Royal Hobart Hospital, Hobart, TAS Australia; 7 Nehme and Therese Tohme Multiple Sclerosis Center, American University of Beirut Medical Center, Beirut, Lebanon; 8 Department of Neurology, Faculty of Medicine, University of Debrecen, Debrecen, Hungary; 9 Department of Neurology, Antwerp University Hospital, Drie Eikenstraat 655, 2650 Edegem, Belgium; 10 Translational Neurosciences Research Group, Faculty of Medicine and Health Sciences, University of Antwerp, Universiteitsplein 1, 2610 Wilrijk, Belgium; 11 Faculty of Medicine, Isfahan University of Medical Sciences, Isfahan, Iran; 12 Neurology, Dr. Etemadifar MS Institute, Isfahan, Iran; 13 Izmir University of Economics, Medical Point Hospital, Izmir, Turkey; 14 Multiple Sclerosis Research Association, Izmir, Turkey; 15 Department of Neurology and Center of Clinical Neuroscience, First Faculty of Medicine, Charles University in Prague and General University Hospital, Prague, Czech Republic; 16 Department of Neurology, School of Medicine, Koc University Research Center for Translational Medicine (KUTTAM), Koc University, Istanbul, Turkey; 17 College of Medicine & Health Sciences and Sultan, Qaboos University Hospital, Sultan Qaboos University, Al-Khodh, Oman; 18 Department of Neuroscience, Hospital Germans Trias I Pujol, Badalona, Spain; 19 Department of Neurology, Universitary Hospital Ghent, Ghent, Belgium; 20 Department of Medical and Surgical Sciences and Advanced Technologies, GF Ingrassia, Catania, Italy; 21 Multiple Sclerosis Unit, AOU Policlinico G Rodolico-San Marco, University of Catania, Via Santa Sofia 78, 95123 Catania, Italy; 22 Department of Neurology, Medical Faculty, Karadeniz Technical University, Trabzon, Turkey; 23 Department of Biotechnological and Applied Clinical Sciences (DISCAB), University of L’Aquila, L’Aquila, Italy; 24 Department of Neurology, Royal Brisbane Hospital, Brisbane, Australia; 25 University of Queensland, Brisbane, Australia; 26 Department of Neurology, Haydarpasa Numune Training and Research Hospital, Istanbul, Turkey; 27 Hunter Medical Research Institute, University of Newcastle, NeurologyNewcastle, Australia; 28 Hunter New England Health, John Hunter Hospital, New Lambton Heights, NSW Australia

**Keywords:** NMOSD, Seronegative, Progression independent of relapses, Relapse-associated worsening, EDSS, Disability

## Abstract

**Background:**

Previous studies have indicated that progression independent of relapse activity (PIRA) is uncommon in patients with aquaporin- 4 antibody-positive (AQP4-IgG) neuromyelitis optica spectrum disorder (NMOSD). However, the patterns of disability accumulation in seronegative NMOSD are unknown. This study aimed to evaluate the prevalence of PIRA and relapse-associated worsening (RAW) in seronegative NMOSD.

**Methods:**

We conducted a retrospective, multicentre cohort study of seronegative NMOSD patients from the MSBase registry. Inclusion criteria required at least three recorded expanded disability status scale (EDSS) scores: baseline, progression, and 6 months confirmed disability progression (CDP). For those with 6-month CDP, the presence or absence of relapse between baseline and progression determined the classification as RAW or PIRA, respectively. Descriptive statistics were employed to present the data.

**Results:**

This study included 93 patients, with a median follow-up duration of 5.0 years (Q1 2.8, Q3 8.4). The cohort predominantly consisted of female patients (77.4%), with a median age of onset of 33.9 years (Q1 26.1, Q3 41.2). PIRA was observed in 1 case (1.1%), whilst RAW was documented in 7 cases (7.5%).

**Conclusion:**

This international cohort study confirms that CDP is uncommon in seronegative NMOSD. Given more than three quarters of CDP occur due to RAW, therapeutic strategies should focus primarily on preventing relapses.

**Supplementary Information:**

The online version contains supplementary material available at 10.1007/s00415-025-13064-6.

## Introduction

Neuromyelitis optica spectrum disorder (NMOSD) is a rare inflammatory disease of the central nervous system (CNS), with an estimated prevalence of 1.51 per 100,000 individuals [1, 2]. Whilst most cases are associated with pathogenic aquaporin- 4 antibodies (AQP4-IgG), approximately 20% of cases are seronegative [2]. Although seronegative NMOSD is a heterogeneous disease and carries risks of misdiagnosis with related conditions such as multiple sclerosis (MS), recent studies where neuroimmunology experts classified patients have identified that both AQP4-IgG positive and seronegative subtypes of NMOSD exhibit comparable relapse rates and responses to conventional treatments [3, 4].

Most patients with AQP4-IgG positive NMOSD do not experience confirmed disability progression (CDP); for those who do, only a small proportion is due to progression independent of relapse activity (PIRA) [5]. Consequently, the primary therapeutic goal in AQP4-IgG positive NMOSD is the prevention of relapses to mitigate relapse-associated worsening (RAW). However, data on the patterns of disability progression in seronegative NMOSD are limited. In this study, utilising the MSBase data registry, we aimed to assess the rates of PIRA and RAW in patients with seronegative NMOSD.

## Methods

### Study design

This was a retrospective cohort study of seronegative NMOSD patients enrolled in the MSBase Neuroimmunology Registry (ACTRN12605000455662), which contains patients from 174 centres and 44 countries with neuroinflammatory conditions, followed in expert centres. The follow-up period spanned from the initial to the most recent patient visit, with data extraction conducted in November 2022.

### Ethics

Ethical approval was obtained from the Alfred Health Human Research Ethics Committee (HREC 528/12), as well as from local ethics committees at all participating centres, unless exempted by local regulations and laws. All participants provided informed consent, in accordance with the Declaration of Helsinki [6].

### Participants

**Fig. 1:**
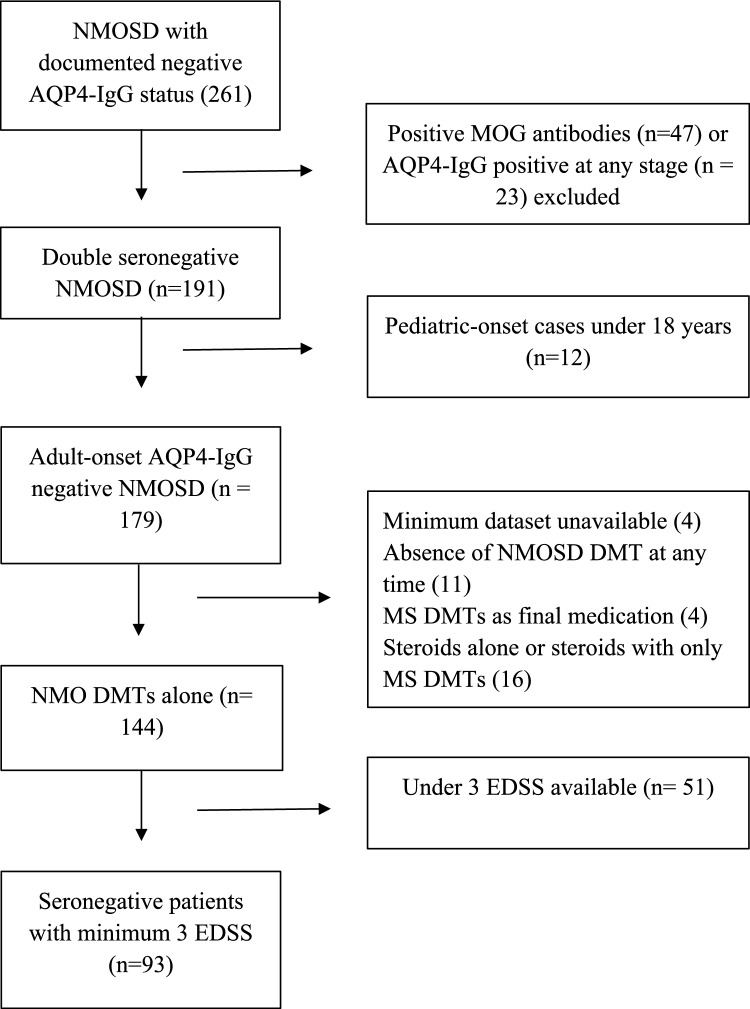
Inclusion criteria in seronegative NMOSD. *AQP4-IgG* aquaporin- 4 antibody, *MS* multiple sclerosis, *NMO* neuromyelitis optica, *NMOSD* neuromyelitis optica spectrum disorder, *DMT* disease-modifying therapy, *MOG* myelin oligodendrocyte glycoprotein, *EDSS* expanded disability status scale

Patients were included in this study if they met the following criteria: diagnosis of seronegative NMOSD according to the 2015 International Panel for NMO Diagnosis (IPND) diagnostic criteria as determined by the treating neuroimmunologist [1], availability of the minimum dataset [6], a minimum of 3 recorded expanded disability status scale (EDSS) scores, and treatment with an NMOSD immunotherapy at last encounter (Fig. 1). Although there are no approved therapies for seronegative NMOSD, based on evidence from the literature [4], off-label NMOSD immunotherapies included azathioprine, rituximab, mycophenolate, mitoxantrone, cyclophosphamide, hematopoietic stem cell transplant, satralizumab, tocilizumab, eculizumab, ravulizumab, and inebilizumab. We required three EDSS scores in order to record a baseline EDSS, a progression EDSS, and a CDP EDSS, at a minimum.

Exclusion criteria encompassed unknown or positive AQP4-IgG status, positive myelin oligodendrocyte glycoprotein (MOG)-IgG serostatus, treatment with an MS immunotherapy at last review, and disease onset before 18 years of age. Assay information for autoantibody testing was available from most of the institutions involved in this study, accounting for 82 patients (88.1% of the cohort), the majority of whom (92.7%) use cell-based assay for AQP4-IgG and MOG-IgG testing.

### Outcomes and definitions

We utilised a roving baseline EDSS approach, where any change in the EDSS score could establish a new reference point, coupled with re-baselining, which involved resetting the EDSS to a new baseline after a CDP. An event was defined as an increase in EDSS within 24 months from a roving baseline: increase of 1.5 if the baseline EDSS was 0, 1 if the score was ≥ 1 and ≤ 5.5, and 0.5 if the baseline EDSS was > 5.5 [7, 8]. CDP was determined in patients with an event and required a recorded EDSS 6–24 months post-event, sustained worsening, and no relapses between the event and confirmation dates. Relapses were clinician reported based on the emergence of new symptoms lasting ≥ 24 h in the absence of infection or pyrexia and occurring more than 30 days after the previous relapse [9].

The primary outcome was the identification of PIRA and RAW. The presence or absence of relapses between the baseline and event EDSS dates in patients with CDP determined RAW and possible PIRA, respectively [8]. Cases identified as possible PIRA required validation by the local neurologist at each respective institution, including confirmation that there was no clinical or radiological evidence of relapse between the baseline and event dates. Sustained worsening for RAW was defined as persistent deterioration in EDSS compared to baseline, whereas for PIRA, no recovery from the event EDSS score was permitted. In a sensitivity analysis, we also examined PIRA cases where partial recovery was allowed, analogous to RAW.

### Statistical analysis

Statistical analyses were conducted using R version 4.2.2. The primary outcome is presented using descriptive statistics as numbers and percentages. Demographic data are described using the median and quartiles for continuous variables, and numbers and percentages for categorical variables.

## Results

Overall, 93 patients were included in our study, with a median follow-up duration of 5.0 years (Q1 2.8, Q3 8.4) (Table 1). All patients met the 2015 IPND diagnostic criteria for NMOSD as determined by the expert neuroimmunologists at each centre, which included exclusion of disease mimics and alternative diagnoses. The cohort was predominantly female (77.4%), with 37.6% of patients exhibiting involvement of the optic pathways and 36.6% showing spinal cord involvement at disease onset. The median age at disease onset was 33.9 years (Q1 26.1, Q3 41.2). Most patients had a relapsing disease course (86.0%) with disease onset prior to 2015 (80.0%).

**Table 1:** Demographic baseline characteristics of seronegative NMOSD patients

Clinical characteristics	Seronegative negative (*n* = 93)
Sex	72 F (77.4%), 21 M (22.6%)
Ethnicity	4 Asians (4.3%), 74 non-Asian (79.6%), 15 unknown
Relapsing disease course	80 (86.0%)
Assay methodology^+^	59 (72.0%) fixed CBA, 17 (20.7%) live CBA, 6 ELISA
Site of disease onset	
Optic pathways only	35 (37.6%)
Spinal cord only	34 (36.6%)
Brainstem only	9 (9.7%)
Multiple sites	8 (8.6%)
Unknown site	7 (7.5%)
Median age at disease onset	33.9 years, Q1 26.1, Q3 41.2
Disease duration (median), years	7.9 years, Q1 4.9, Q3 14.8
Duration of follow-up (median), years	5.0 years, Q1 2.8, Q3 8.4
First recorded EDSS (median)	3, Q1 2, Q3 4.0
Disease onset (year)	
< 2000	12 (12.9%)
2000–2010	33 (35.5%)
2011–2015	29 (31.2%)
> 2015	19 (20.4%)
Treatment (exposure at any time)	
Azathioprine	59 (63.4%)
Cyclophosphamide	12
Satralizumab	1
Copaxone	2
Interferon beta	14
MMF	11
Rituximab	62 (66.7%)
Natalizumab	1
Methotrexate	2
Mitoxantrone	2
Chronic* IVIG	5
Chronic* PLEX	2
Chronic* Steroids	31

*NMOSD* neuromyelitis optica spectrum disorder, *CBA* cell-based assay, *EDSS* expanded disability status scale, *MMF* mycophenolate mofetil, *IVIG* intravenous immunoglobulin, *PLEX* plasma exchange.

*Chronic was defined for these medications as known to be at least 6 months duration. Other exposures to these therapies, or when duration of therapy was not known, were considered to be acute therapies.

^+^Assay information for autoantibody testing was available for 82 patients.

Twenty-four patients experienced 46 relapses that were associated with changes in EDSS meeting the minimal criteria for disease deterioration. Among these, 6 patients (6.5% of the total cohort) had 7 RAW events (7.5%), 4 of whom had no recovery. Thirty-five relapses were associated with complete recovery and 4 relapses were associated with partial recovery not fulfilling criteria for sustained worsening.

Thirteen patients (with 13 incidences) met criteria for possible PIRA. However, only one was confirmed as PIRA (1.1%) after local neurologist review (Table 2). Most data corrections were due to incorrect EDSS recording (9 patients/incidences). Other reasons included EDSS increase due to age-related deconditioning, missing data, and an undocumented relapse. In the sensitivity analysis, where the allowance for partial recovery in RAW was extended to PIRA, no additional confirmed PIRA events were identified (Supplementary Fig. 1).

**Table 2:** Clinical profile of seronegative NMOSD patient with confirmed PIRA

Patient	Serostatus	EDSS change (baseline to event EDSS)	Age at disease onset (years)	Sex	Disease duration (years)	Follow-up time (years)	Treatments	Latest relapse	PI comments
1	Seronegative	EDSS of 3.5 to 4.5 over 9 years	37.7	Female	27.9	17.1	Rituximab, azathioprine	4/10/2010	Gradual step-wise deterioration in the absence of clinical or radiological (MRI) relapses (ambulation functional score). Patient also had vertebral fractures which may have contributed, however is likely to have had PIRA

*NMOSD* neuromyelitis optica spectrum disorder, EDSS, expanded disability status scale, *MRI* magnetic resonance imaging, *MSK* musculoskeletal, *PIRA* progression independent of relapses

## Discussion

This represents the first study to demonstrate the rarity of PIRA (1.1% of the entire cohort) in a large cohort of seronegative NMOSD patients. Whilst disability accrual in seronegative NMOSD is primarily driven by relapses, most patients exhibit at least partial recovery following these events, as reflected in the relatively low prevalence of RAW observed in our cohort.

RAW and PIRA have been extensively characterised in the context of MS; however, the underlying mechanisms of PIRA remain incompletely understood [10]. RAW is considered an inflammatory process caused by new focal lesions. In contrast, PIRA represents a more insidious process (‘smouldering inflammation’) of diffuse white and grey matter injury, compartmentalised inflammation (e.g. secondary lymphoid follicles in the meninges), or expansion of pre-existing lesions (slowly expanding lesions or iron rim lesions) [10, 11]. In MS, neuroaxonal damage is believed to be linked to disease progression [12]. Pathological studies in NMOSD have failed to identify cortical demyelination, global neurodegeneration, deep grey matter involvement, or meningeal lymphoid follicle-like infiltrates [13]. These pathological differences may account for the apparent scarcity of clinically evident PIRA in AQP4-IgG positive NMOSD [2]. The pathophysiological mechanisms underlying seronegative NMOSD remain poorly characterised but are likely distinct from those of AQP4-IgG positive NMOSD. Serum neurofilament light chain, a biomarker of neuroaxonal injury, has been reported at higher levels in seronegative NMOSD compared to AQP4-IgG positive NMOSD [3]. In contrast, cerebrospinal fluid concentrations of glial fibrillary acidic protein (GFAP) are significantly elevated in AQP4-IgG positive NMOSD relative to seronegative NMOSD, strongly suggesting that the latter is not primarily an astrocytopathy [14]. These pathophysiological differences, along with disease heterogeneity and the risk of MS misdiagnosis, led us to hypothesise that PIRA would be more common in seronegative NMOSD than in seropositive NMOSD. However, the number of PIRA events in the present study was similarly low to our recent study examining the patterns of disability accrual in AQP4-IgG NMOSD [5]. Whilst this finding provides reassurance against misclassification of MS cases as seronegative NMOSD in expert centres, it also emphasises the need for comprehensive research into seronegative NMOSD.

Despite the paucity of reports of clinically apparent PIRA in NMOSD, subclinical progression has been reported in optical coherence tomography (OCT) studies with reduced ganglion cell inner plexiform (GCIP) layer thickness in NMOSD patients without optic neuritis during longitudinal follow-up. Visual evoked potential (VEP) studies with longitudinal follow-up have also shown prolongation of P100 latencies in patients without known optic neuritis [15–19]. In many of these studies, there was a history of optic neuritis in one eye, and one proposed explanation for the changes in the contralateral eye relate to trans-synaptic degeneration involving the optic radiations and subsequently involvement of the contralateral eye [15, 20]. However, thinner GCIP layer measurements have also been reported in NMOSD patients compared to healthy controls without a prior history of optic neuritis, suggesting clinically unrecognised optic neuropathy or subclinical retinal damage could be responsible [19]. The latter has been investigated in a multi-centre study that found no evidence of retinal atrophy outside of relapse, suggesting outer retinal damage occurs during the active phases of the disease [21, 22]. Cervical cord atrophy has also been observed in NMOSD patients without prior visible lesions on MRI [23]. Whilst it is possibly due to microscopic inflammation not visible on standard MRI, it may also represent neurodegeneration. A report of progressive myelopathy in a newly diagnosed NMOSD patient suggested primary progression, however, was more likely a protracted course of relapse-associated worsening due to delayed treatment [24]. Moreover, some studies have reported cognitive impairment, primarily lower memory and attention scores, in 29–70% of patients with NMOSD [25–27]. Cognitive impairment may result from diffuse cortical neuronal loss following a relapse or disruption to synaptic plasticity regulated by astrocytic AQP4 expression [28, 29]. Although cognitive dysfunction may indicate disease progression, cognitive assessment may be confounded by other factors common in NMOSD, such as increasing age, depression, sedating medications, pain, and sleep disturbances [26, 27]. Interestingly, there have been reports of cognitive improvement following complement-targeted therapy, highlighting the potential role of inflammation in cognitive impairment [30]. Limited longitudinal biomarker studies in AQP4-IgG positive NMOSD have produced conflicting results, with one study finding increases in sGFAP in patients without baseline attack [31], whilst another study of individuals treated with high-efficacy therapy did not show any substantial sGFAP changes during the inter-attack period [32]. Given the discrepancy in reported rates of PIRA in clinical versus paraclinical studies, larger studies with longer follow-up are required. Notably, most existing studies have focused exclusively on AQP4-IgG positive NMOSD, and the potential for subclinical progression in seronegative NMOSD remains an area requiring further investigation.

Historically, seronegative NMOSD was considered a less relapsing and more benign disease as compared to AQP4-IgG positive NMOSD [1]. Recent evidence from similar studies involving neuroimmunology experts, however, suggests comparable risks of relapses in both subtypes of NMOSD [4]. In addition, one study identified that seronegative NMOSD patients reach disability milestones (EDSS 4, 6, and 7) more rapidly and with fewer relapses than seropositive patients [33]. We demonstrate similarly low rates of RAW in our seronegative cohort as we did in our AQP4-IgG positive cohort (7.5% vs 7.2%) [5]. Although the limited number of RAW events in our study precluded an analysis of factors associated with post-relapse recovery, other studies in AQP4-IgG positive NMOSD suggest that effective acute and maintenance immunotherapy is critical in facilitating post-relapse recovery [33, 34]. Whilst the prevalence of RAW may be lower than anticipated, RAW events accounted for the majority of CDP cases (7/8; 87.5%) and a sizable proportion of patients with relapses meeting the minimum requirements to be classified as an event experienced RAW (6/24; 25%), highlighting the importance of relapse prevention.

The strengths of this study include the stringent inclusion criteria and diagnosis of seronegative NMOSD by neuroimmunology experts in tertiary or quaternary centres, the large cohort size, and the rigorous verification process required for PIRA identification. However, our study is limited by its reliance on overall EDSS changes as the sole measure of disability, which primarily reflects changes in mobility. Future research should incorporate additional clinical assessments such as 9-hole peg test, 25-foot walk test, symbol digit modality tests, and comprehensive cognitive assessments, as well as paraclinical tests such as OCT and VEP. This is particularly relevant in NMOSD, where severe visual impairment can result from optic neuritis episodes, and subclinical progression has been reported in some studies. Additionally, the MOG antibody status was not documented in all cases as the majority of patients had disease onset prior to the availability of MOG antibody testing [35]. Whilst individuals with a confirmed positive MOG serostatus were excluded, it remains uncertain whether any of the included patients would have tested positive had the test been accessible at the time of diagnosis. This represents an inherent limitation in studies investigating seronegative NMOSD cases predating 2018, when MOG antibody testing became commercially available, and 2023, when the first diagnostic criteria for MOG antibody-associated disease (MOGAD) were established [36]. Another limitation of this study is the lack of baseline MRI data. However, the absence of radiological activity in the brain and spinal cord was a prerequisite for patients with confirmed disability progression prior to PIRA diagnosis. This approach was chosen as the primary objective of this study was to identify cases of PIRA.

## Conclusion

This study confirms the rarity of PIRA and relatively low incidence of RAW in seronegative NMOSD patients treated with immunosuppressive therapies. Although most patients recover at least partially from relapses in the contemporary setting, relapse prevention should continue to be the goal of management in NMOSD.

## Supplementary Information

Below is the link to the electronic supplementary material.

Supplementary file1 (DOCX 35 KB)

## Data Availability

Data may be available upon reasonable request from university or research institute affiliated academics. Data are not available to commercial entities. The data contained in this manuscript are the property of the individual contributing centres listed in authorship affiliation statements, and permission from each will need to be sought. Data access requests may be conditional on obtaining approvals from the appropriate institutional review boards at each contributing centre. The MSBase registry is a data processor and warehouses data from individual principal investigators who agree to share their datasets on a project-by-project basis. Therefore, as a data processor, the MSBase Registry cannot grant direct data access. To make a request for data access, please contact the corresponding author (Mastura Monif) for further instructions.
