# Supplementary material for: Progression independent of relapse activity and relapse-associated worsening in seronegative NMOSD: an international cohort study
Source: J Neurol. 2025 Apr 14;272(5):339. doi: 10.1007/s00415-025-13064-6 (PMC11996963; doi:10.1007/s00415-025-13064-6)
Supplement: Supplementary file 1 — Supplementary file1 (DOCX 35 KB) [file 415_2025_13064_MOESM1_ESM.docx]

**Supplemental figure 1: Cases of RAW and PIRA in seronegative NMOSD**

Criteria for RAW fulfilled (7)

Relapse-associated EDSS deteriorations (46)

Partial recovery from event score (0)*

No recovery from event score (1)

Criteria for PIRA fulfilled (1)

Possible PIRA (13)

No recovery from event score (4)

Partial recovery from event score (3)

Complete recovery to at least baseline (35)

Incomplete recovery (4)

Incorrect EDSS (9), missing data (1), undocumented relapse (1), alternative reason (1)

EDSS: expanded disability status scale; RAW: relapse-associated worsening; PIRA: progression independent of relapse activity.

*1 possible PIRA were identified if partial recovery was permitted, however on confirmation with local neurologist these were due to incorrect EDSS recordings.
